# An experimental test of risk perceptions under a new hurricane classification system

**DOI:** 10.1038/s41598-025-14170-1

**Published:** 2025-08-19

**Authors:** Jantsje M. Mol, Nadia Bloemendaal, Hans de Moel, Dianna Amasino, Jennifer M. Collins

**Affiliations:** 1 Center for Research in Experimental Economics and Political Decision Making (CREED), University of Amsterdam, Amsterdam, The Netherlands; 2 Tinbergen Institute, Amsterdam, The Netherlands; 3 Institute for Environmental Studies, Vrije Universiteit Amsterdam, Amsterdam, The Netherlands; 4 The Royal Netherlands Meteorological Institute (KNMI), De Bilt, The Netherlands; 5 Department of Social Psychology, Tilburg University, Tilburg, The Netherlands; 6 School of Geosciences, University of South Florida, Tampa, USA; 7 Present Address: Rotterdam University of Applied Sciences, Rotterdam, The Netherlands

**Keywords:** Psychology and behaviour, Environmental economics, Climate-change adaptation

## Abstract

During a hurricane, it is vital that individuals receive communications that are easy to process and provide sufficient information to allow informed hurricane preparedness decisions and prevent loss of life. We study how different hurricane warning scales, the traditional Saffir-Simpson Hurricane Wind Scale (SSHWS) versus the newly developed Tropical Cyclone Severity Scale (TCSS), impact intent to evacuate and understanding of hurricane severity. We use a between-subject design where participants are assigned to either the traditional SSHWS or the new TCSS scale. We collected data in a large-scale (~ 4000 participants) online experiment to examine potential differences in comprehension, risk perception, anticipated evacuation, and preparation decisions among residents in U.S. coastal states prone to hurricanes. We find that participants using the TCSS scale are better at identifying the main hazard of a hurricane. For evacuation, the TCSS leads to significantly higher evacuation intent as opposed to SSHWS in cases where the TCSS is at least two categories higher (due to rainfall or storm surge being the main hazard rather than wind). In addition, the TCSS also seems to have a positive effect on taking appropriate precautionary measures, though not always at our stated significance level. Overall, our results demonstrate that people make better informed and more appropriate decisions with the TCSS as opposed to the currently used SSHWS.

*Protocol Registration* The stage 1 protocol for this Registered Report was accepted in principle on 14 October 2024. The protocol, as accepted by the journal, can be found at: https://doi.org/10.17605/OSF.IO/AYXTK. The approved Stage 1 protocol is available here: https://osf.io/m3swr.

**Supplementary Information:**

The online version contains supplementary material available at 10.1038/s41598-025-14170-1.

## Introduction

Landfalling hurricanes can lead to casualties and considerable damages through strong winds, high storm surges, and heavy rainfall. Risk communication is often done through use of the Saffir-Simpson Hurricane Wind Scale (SSHWS), which categorizes a hurricane’s maximum 1-min sustained wind speed on a scale of 1–5^1^, and uses the terms “Tropical Storm” and “Tropical Depression” for weaker storms. Yet, recent research shows that storm surge (49%) and rainfall (27%) are the main causes of fatalities for U.S. hurricanes, compared to only 8% of wind-related fatalities^2^. Classifying hurricane threats solely based on wind speed therefore does not capture its full severity. One striking example of an event where the SSHWS category mismatched the severity of the event, was Hurricane Katrina^3^ in 2005. Katrina ranked as a Category 3 on the SSHWS, yet caused over 1,800 casualties and US$125 billion damage due to extreme flooding following high storm surge and rainfall totals. There are numerous examples of tropical storms and hurricanes that have created significant impacts from rainfall or storm surge flooding while being a low-category (i.e., lower than Category-3) storm. For instance, Hurricane Sandy, a post-tropical storm upon landfall in 2012, exhibited the potential for Category 1 storms to produce significant storm surge, reaching up to 3.9 m with this particular storm^4^. Likewise, Hurricane Florence made landfall as a Category 1 hurricane in South Carolina in 2018 causing devastating freshwater flooding across the southeastern United States, resulting in 55 fatalities^5^. These types of events are particularly worrisome as it has been found that the category of a hurricane influences the decision to evacuate^6^.

Providing adequate information on a pre-event basis is critical to encourage proper decision making, such as evacuating or taking protective measures^7^. Without satisfactory and complete information, an individual is likely to miscalculate their personal risk or even potentially be moved to inaction^8,9^, a process also known as milling. This means that a person spends time trying to gather more complete information before continuing with their decision-making during a disaster, which can lead to valuable time loss, worsening the individual’s risk due to inaction^9^. Moreover, most people incorrectly interpret the SSHWS by thinking that hurricane damage potential increases linearly with the category^8^. Recent studies have shown an over-reliance of the SSHWS by the general public^10–13^ despite the fact that the scale does not reflect the full (potential) severity of an event.

Risk perception, or the subjective judgement one makes about risks, is often skewed due to its inherent personal nature^13^. People may not always identify the most pressing threats, which is known as the “risk perception gap” ^14–16^. To overcome the fallibility of the SSHWS and to minimize the risk perception gap, several studies have proposed alternative categorization and indexing methods^17,18^. There are two important caveats with these proposed alternative categorizations; firstly that they often do not reflect the full severity of an event, and secondly that their use as a forecasting tool has not been tested among the general public for effects on risk perception.

To include the three main hazards (wind, rain, storm surge), and as such reflect the full severity of an event, the Tropical Cyclone Severity Scale (TCSS) has been developed^19^. The TCSS is the first alternative pre-event scale to include all three major hazards in its classification and preserves the discrete nature of categorization as used in the SSHWS, thereby maintaining familiarity amongst the general public. The TCSS aims to be easily usable and interpretable, while conveying more information than just wind hazards. The TCSS categorizes all three hazards on a scale of 1 to 5, where the wind categorization is equal to the SSHWS categorization, and the rain and storm surge hazards are categorized using similar criteria as was used for the initial design of the SSHWS. These hazard-based categories are then combined into one final category using three constraints: (i) final category is never lower than the highest hazard-based category, (ii) two major (3 +) hazards of the same category results in a final category of one higher, (iii) a maximum final category of 6 is used when there are either two category 5 hazards, or two category 4 hazards and a category 5 hazard^19^. In cases where wind speed is the main hazard (and rainfall and storm surges hazards thus have a lower category), the TCSS and SSHWS will always yield—by design—the same category.

An advantage of using the SSHWS over another classification method is that it is very simple to use for forecasters and decision-makers, being solely based on wind. Besides, the SSHWS scale has been used for decades making the general public very familiar with it. In addition, the wind speed categories were chosen such that they reflect on the potential damage: category 3 wind speeds are equivalent to “devastating damage”, category 4 and 5 equate to “catastrophic damage”^20^. However, as pointed out before, the SSHWS does not reflect on the rain or storm surge hazard and the associated potential damage, which is why the TCSS was designed. TCSS includes these other two main hazards in a similar way as SSHWS does for wind in order to maintain the categorical nature people are familiar with.

Previous research has shown that the TCSS outperforms SSHWS in predicting damage of historical events^19^. Here, we want to take the next step, which is exploring its potential on future human behavior, including intent to evacuate. We collected data among a large (~ 4000 respondents) sample of relevant decision-makers (residents in U.S. coastal states prone to hurricanes) to examine which scale leads to higher understanding of the main hazard and subsequent action (evacuating, preventive measures). In particular, we were interested in whether better identification of the main hazard would improve appropriate precautionary measures and whether the overall category difference would increase evacuation intent for more dangerous storms. To minimize chances of familiarity, instead of using historical events in our analysis, we instead use a hypothetical (i.e., has not occurred before, but could theoretically occur) storm track from the STORM dataset^21^. Along this storm track, we perturb ten different hurricane conditions (maximum wind speed, storm surge height, and rainfall totals). These hurricane conditions are comparable to historical events in terms of the combination of hazard categories on the TCSS scale. This set-up ensures usage of plausible category combinations, without raising potential familiarity amongst our subject group. Table 1 gives an overview of the scenarios.

**Table 1:** Overview of scenarios.

Name	Wind (mph)	Surge (feet)	Rain (inches)	Category SSHWS	Category TCSS	Historical example	Main hazard
Chi	120 (3)	8 (2)	24 (4)	3	4	Irma (2017)	Rainfall
Lambda	70 (0)	4 (1)	8 (1)	0	1	Gordon (2018)	Rainfall/Storm surge
Omega	100 (2)	4 (1)	20 (3)	2	3	Alex (2010)	Rainfall
Nu	85 (1)	10 (4)	31 (5)	1	5	Florence (2018)	Rainfall
Rho	145 (4)	20 (5)	8 (1)	4	5	Emily (2005)	Storm surge
Sigma	100 (2)	4 (1)	8 (1)	2	2	Bertha (1996)	Wind
Tau	120 (3)	8 (2)	8 (1)	3	3	Fran (1996)	Wind
Phi	120 (3)	20 (5)	12 (2)	3	5	Katrina (2005)	Storm surge
Theta	145 (4)	20 (5)	31 (5)	4	6	Wilma (2005)	Rainfall/Storm surge
Psi	120 (3)	4 (1)	31 (5)	3	5	Sally (2020)	Rainfall

We list values in U.S. customary units, which is how they were stated in the experiment, since our target group is familiar with this unit system. Wind, surge, and rain columns indicate values in mph, feet and inches respectively, followed by the individual TCSS category in parentheses. Note that Hurricane Theta was slightly different in the pilot (wind speed 154 mph/category 5).

Using these scenarios with either the SSHWS or TCSS scale presented with text-only or text and a graphic for an individual respondent, we test the following hypotheses:

### H_1_

Participants exposed to TCSS warnings answer more quiz questions about the main hazard of the hurricane correctly than participants exposed to warnings in SSHWS format (sum of correct quiz questions across scenarios).

### H_2A_

Participants exposed to TCSS warnings express more evacuation intent than participants exposed to warnings in SSHWS format for scenarios where the highest TCSS category is at least two (category) points higher.

### H_2B_

Participants exposed to TCSS warnings express similar evacuation intent in scenarios that are the same category as SSHWS.

### H_3A_

Participants exposed to TCSS warnings select more relevant precautionary measures related to non-wind hazards (i.e. flood shields or sandbags) than participants exposed to warnings in SSHWS format for scenarios where the highest TCSS category is at least two (category) points higher.

### H_3B_

Participants exposed to TCSS warnings will select equal precautionary measures related to wind to those shown SSHWS warnings.

Our pilot study (N = 401) conducted in August 2023 provided initial support for the formulated hypotheses (see Pilot Data in Supplementary Information and Methods for more details). In addition to the hypotheses specified above, we explore the impact of various (mostly confounding) variables on the intent of taking action (evacuation and precautionary measures). Specifically, these include:

- Text only versus text and a graphic. We expect H1-3 to hold regardless of presentation format, but we test whether graphics impact any outcomes and whether there is any interaction between scale and graphics on outcomes.
- Prior hurricane experience. We expect this to have a positive effect on participant’s intent on taking action.
- Near-miss event experience. We expect this to have a negative effect on participant’s intent on taking action.
- Identification of the main hazard. We expect this to be a mediator, with a positive effect on participant’s intent on taking action appropriate for that hazard.
- Risk perception. We expect this to be a mediator, through which participants take action.
- Measures of information-gathering: clicking for additional information, time spent on additional information, and overall response time on page. We test whether these measures mediate accuracy on quiz questions.

In summary, the current work provides an investigation of how residents respond to hurricane warnings and whether a different type of hurricane categorization scale can help residents in correctly identifying the hazards they may be exposed to and trigger more appropriate action. This knowledge will help forecasters and emergency services to improve their communication of threatening situations to citizens and as a result make society more resilient to hurricanes.

## Methods

### Ethics information

This research complies with the ethical regulations for research conducted with human participants. The study protocol has been approved by the ethics review board of the Economics and Business Ethics Committee, University of Amsterdam (reference #EB-5573). Informed consent was obtained from all respondents prior to participation. Participants received a flat payment for their time spent participating and on top of that they could earn additional bonus payments depending on their accuracy in answering the questions in the study (see Design for details).

### Design

**Fig. 1:**
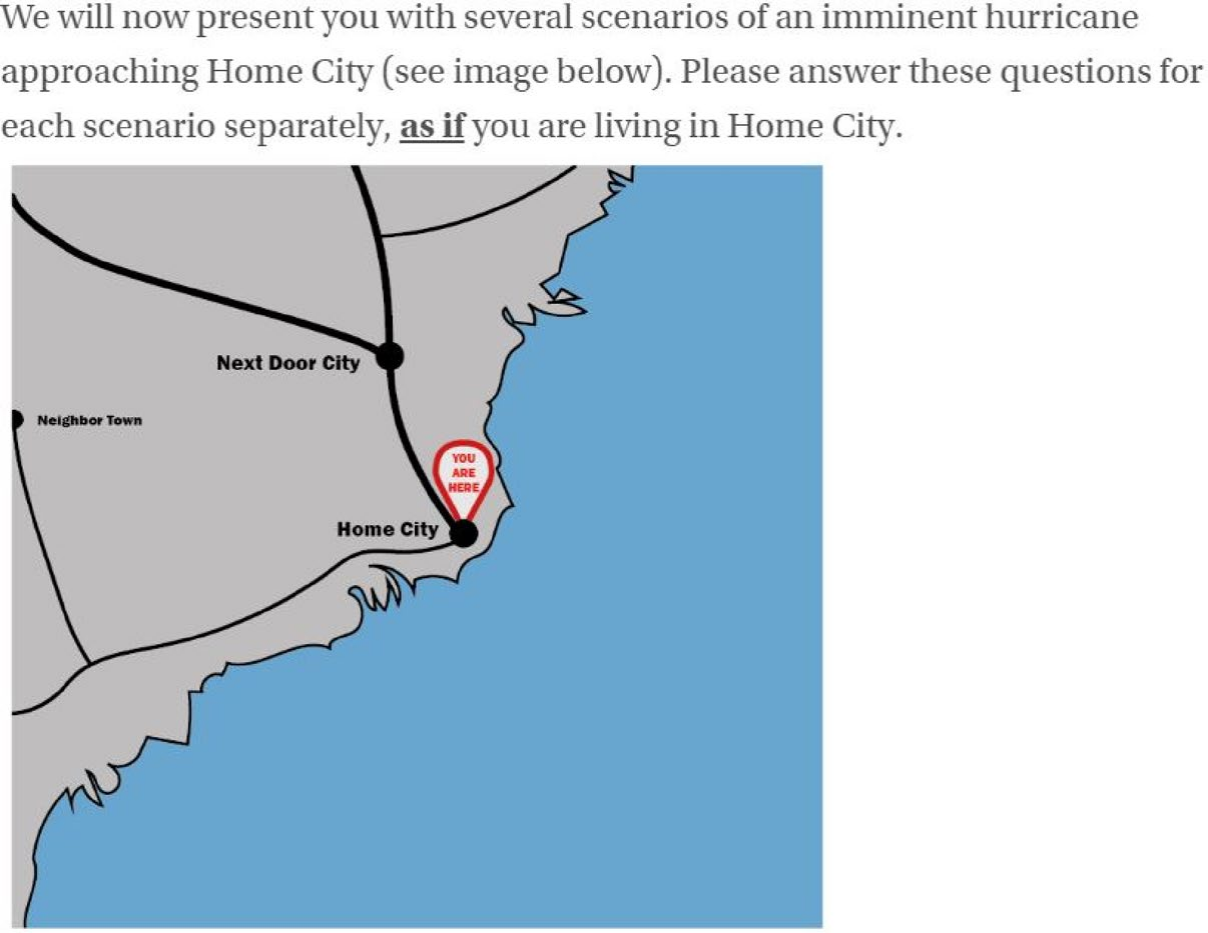
Introduction to the hypothetical scenarios for respondents. Image created in Adobe Illustrator (version 27.1) by the authors.

Participants accessed the survey through an anonymous link at the Prolific platform. We did not store IP-addresses. After consenting, participants reported the U.S. state and county in which they spend most of their time between June and November (i.e. the official Atlantic hurricane season). Inhabitants of inland states were screened out at this stage. The target group proceeded with the introduction of our hypothetical scenarios (see Fig. 1). As an attention check, we asked participants which city is located at the you-are-here arrow (Next Door City/Neighbor Town/Home Town/Home City/Next Door Town/Neighbor City). Participants who answered incorrectly were warned. Participants who answered incorrectly twice were screened out. Those who passed the attention check were randomly divided into four treatment groups (2 × 2 between-subjects design: scale (TCSS versus SSHWS) by information type (graphic versus text-only) and presented with 10 hypothetical scenarios (see Table 1) in random order. The main treatment of interest is the scale, but information type is included to be able to control for the effect of graphics.

**Fig. 2:**
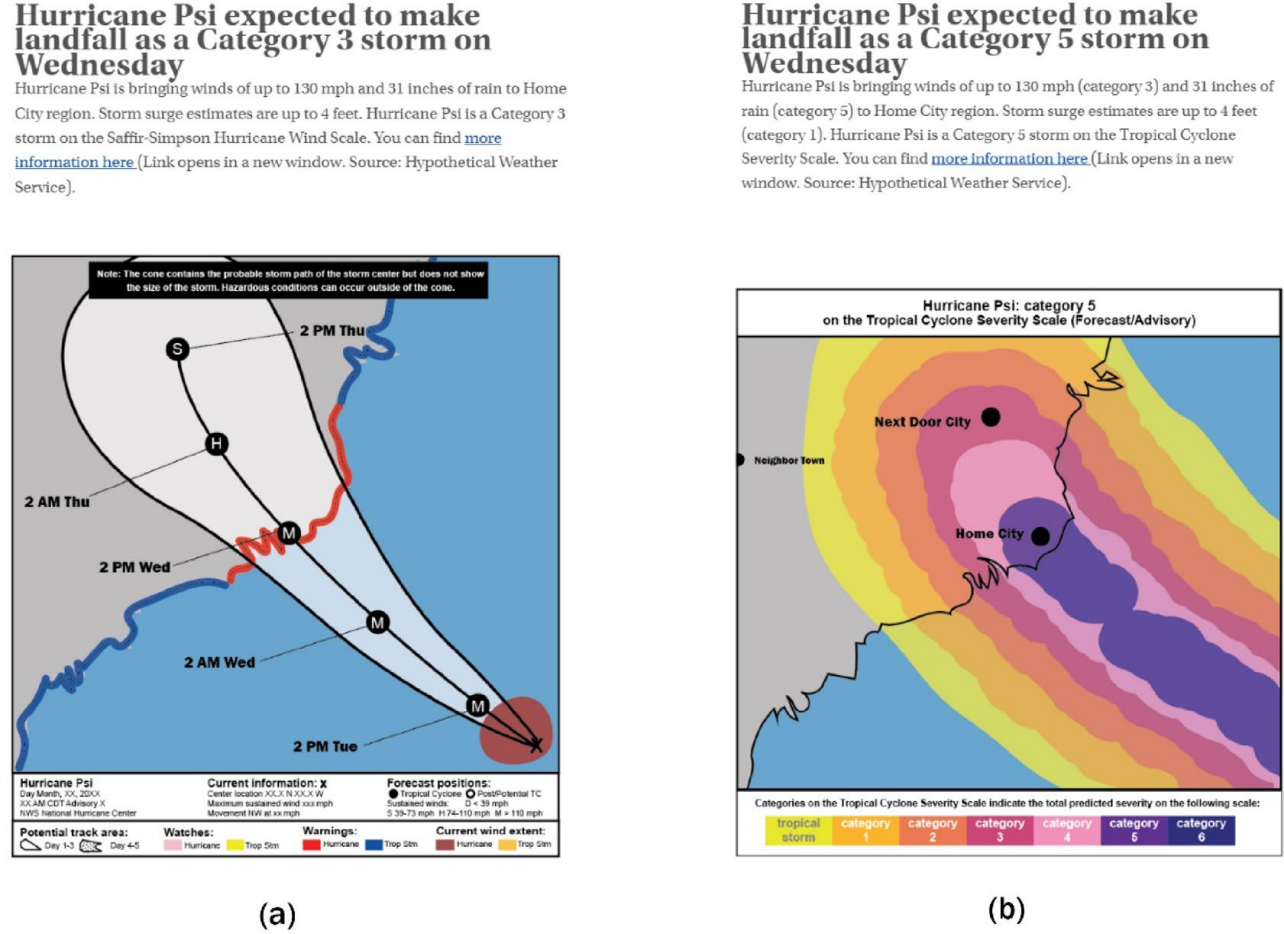
Screenshot of treatments. Left panel: SSHWS treatment. Right panel: TCSS treatment. In the text-only treatment, participants were not presented with the graphics. Across all treatments, participants could click the *more information here* button, which lead to a pop-up screen with more information on each of the hazards. Images created in Adobe Illustrator (version 27.1) by the authors.

Each scenario contains a hurricane warning with a picture (graphic treatments only) and a link to more information (See Fig. 2). The text and possible picture are geared towards either the SSHWS scale, or TCSS scale (between-subjects design). The information given to the participant is the same for both SSHWS and TCSS, with only the categorization information different. As such, the SSHWS warning also includes information on the height of the storm surge and amount of rain expected, but this does not affect the category of the storm given. For example: *Hurricane Psi is bringing winds of up to 130 mph and 31 inches of rain. Storm surge estimates are up to 4 feet.* For the SSHWS, we are using the visualization depicting the cone of uncertainty, which is the graphic most often used by the National Hurricane Center (NHC)^22^. NHC does use various other graphs, as can be seen in the graphics archive of past hurricanes, such as Hurricane Idalia^23^. These visualizations are also sometimes used in their communication on social media channels such as Facebook or X (https://twitter.com/NHC_Atlantic), and are also used in our experiment behind a ‘more information here’ link.

For the TCSS, both overall category, as well as category numbers for the three individual hazards are given in the text (in parentheses). For example: *Hurricane Psi is bringing winds of up to 130 mph (Category 3) and 31 inches of rain (Category 5). Storm surge estimates are up to 4 feet (Category 1).* Both warnings contain a link to more information. The extra information behind this link (three more panels of figures) is identical for both treatments, showing storm surge, wind, and rainfall forecasts, similar to the information on the NHC website during hurricane season (https://www.nhc.noaa.gov/). Each scenario was followed by a quiz question (“*What is the main hazard of Hurricane X in Home City”*), a question on risk perception^24^ (“*How risky do you think it would be for you to stay in Home City under this situation?”*—on a 5-point Likert scale from not at all risky to extremely risky), evacuation intent^24^ (“*How likely are you to evacuate/leave Home City under this situation?—*on a 5-point Likert scale from very likely to stay to very likely to go)” ) and precautionary measures (“*Would you plan to take any measures to protect your home from this hurricane?*”—tick boxes on window protection, sandbags, moving contents to higher floors and an open box for other answers). Although research on risk perception has indicated that comprehensive scales can identify multiple separable components of risk, such as affect and probability estimation, this was beyond the scope of our investigation^25^. We chose to use simple metrics from the literature for their clarity and brevity because they were asked after each of the 10 scenarios and we prioritized surveying a range of behavioral responses and risk while minimizing participant fatigue. The measures chosen represent some of the most common emergency measures (sandbags and elevating possessions in case of flooding, window protection in case of wind) as apparent from post-disaster surveys^26,27^. After the 10 scenarios, participants answered a follow-up question on evacuation (in case evacuation intent was (very) likely to evacuate at least once: “*If you decide to evacuate, where would you plan to evacuate to?*”—Friends or family/Hotel/Shelter/Other, namely…) or refusing evacuation (“*If you would not evacuate, why not?*”—open answer).

The survey ended with some questions on hurricane preparedness (home type, generator, cellar, insurance), risk perception (distance to coastline, living in flood zone, previous experience with hurricanes) and some demographics (age, level of education, homeowner, gender, income, disabilities in household). All surveys with all measures and items can be found in our OSF repository (https://osf.io/d4pje/). The average time to complete a survey was 16 min (sd = 10).

### Scenario development

The set of scenarios was specifically designed to explore the full range of hurricanes from high to low intensities, and a range of differences in intensities between TCSS and SSHWS. To achieve this, we used the following set of criteria:

1. A relatively equal distribution of the main hazard (i.e. rainfall, wind and storm surge) for the storms;
2. Include high-category events as well as low-category events, both for the SSHWS and TCSS;
3. Include events where the difference between the SSHWS and the TCSS is equal to two or more category points, as well as events where this difference is smaller;
4. To ensure realistic combinations of the hazards, each of our scenarios should have a historical counterpart (note that the counterpart is not communicated in the experiment).
5. To make the distribution of events in terms of their SSHWS and TCSS category differences realistic, we follow a similar distribution as was found in the paper that introduced TCSS^19^ for the historical events (see main text below);
6. Include one Category-6 scenario (for TCSS) to test perception towards this new category

We used Greek names for our hypothetical hurricanes that have never been used before as hurricane names to take away potential associations with past hurricanes. In addition, to further reduce chances of potential association, instead of using a historical hurricane as underlying data for our maps, we used a hypothetical (synthetic) hurricane track. This synthetic hurricane is extracted from the STORM dataset^21^, a dataset composed of 10,000 years of synthetic hurricane activity. This way, we ensured working with a realistic, hypothetically possible hurricane, without using an *actual* historical hurricane that participants may have familiarity with. Wind speed information is initially taken from STORM, but perturbed around the peak intensity to end up at different categories corresponding to our 10 storms based on historical counterparts. Wind footprint maps are created by fitting a 2D parametric wind field model^28^ to the synthetic track. For peak storm surge and rainfall, we chose arbitrary values corresponding to the respective TCSS category threshold values^19^. We assume that the peak storm surge occurs at the location of landfall and decays equally towards both sides of the track. Similarly, we assume that the peak rainfall occurs near the location of peak wind intensity, and decays similarly to the wind decay. Table 1 gives an overview of the scenarios and their respective categories and historical counterparts. By criterion 5, we closely follow the distribution of category increments as was found in Fig. 2 of the original paper^19^. We do this to ensure the participants are presented with a realistic set of category changes, i.e., that our set is not biased towards either strong category increments on the TCSS as opposed to the SSHWS, or towards minimal differences between the TCSS and SSHWS category. From the population of landfalling hurricanes between 1996 and 2018 examined when TCSS was introduced^19^, 30% did not change in final category, 39% incremented one final category point, 22% incremented 2 points, and 9% incremented 3 or more points. In the sample shown in Table 1, we have 20% of the cases having no change in category, 40% increasing by 1 point, 30% increasing by 2 points and 10% increasing by 3 or more points.

## Results

### Participants

**Fig. 3:**
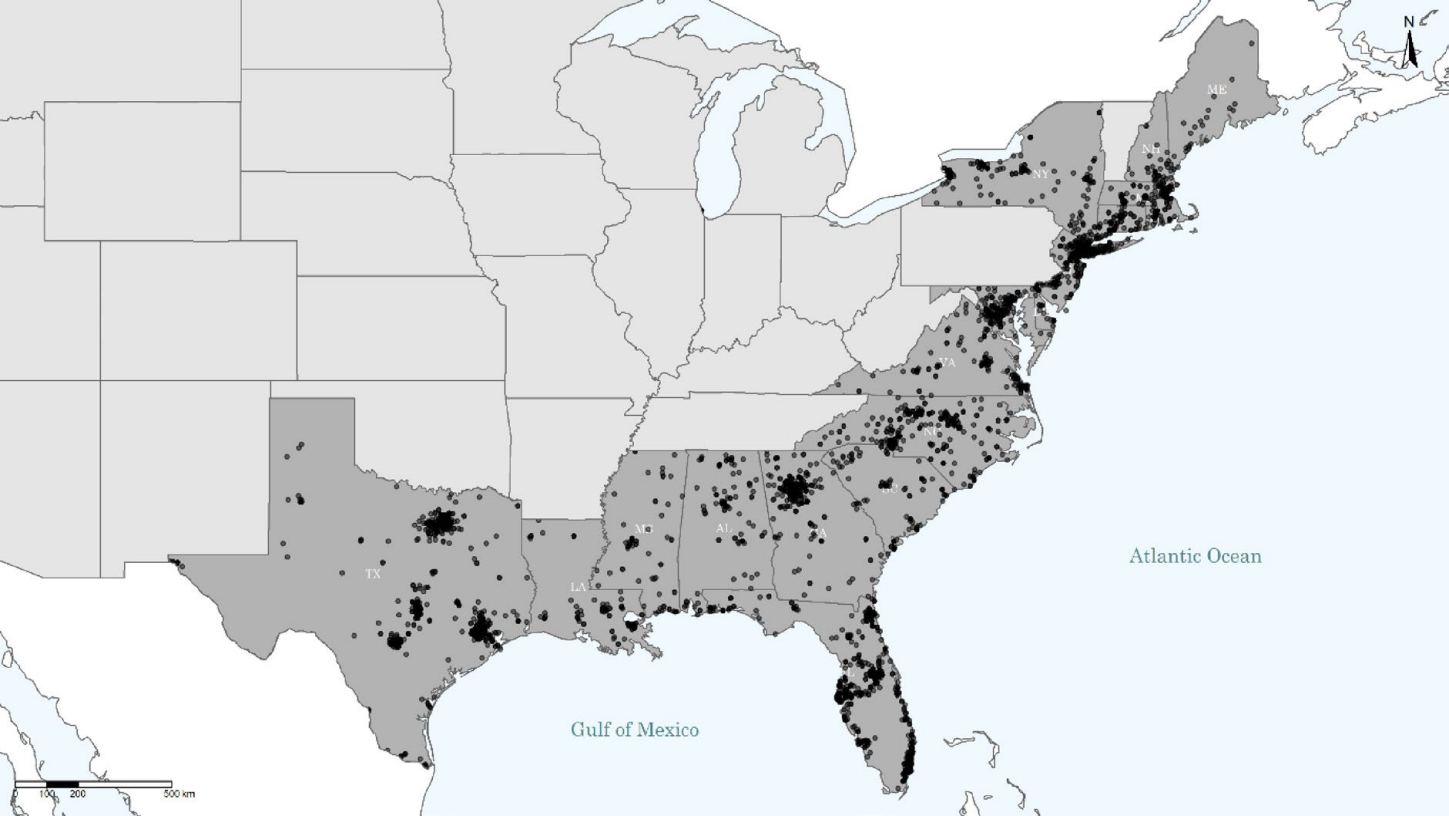
Map of respondents. Each dot indicates the location of a respondent, based on IP-address. Dark grey areas indicate coastal states, where coastal refers to the ocean coast (Alabama, Connecticut, Delaware, Florida, Georgia, Louisiana, Maine, Maryland, Massachusetts, Mississippi, New Hampshire, New Jersey, New York, North Carolina, Rhode Island, South Carolina, Texas, Virginia and Washington, DC).

We recruited a sample of 4360 participants who are U.S. residents living in states under hurricane threat using the Prolific online research platform (https://prolific.co). We targeted respondents in states along the Gulf and East Coast (Alabama, Connecticut, Delaware, Florida, Georgia, Louisiana, Maine, Maryland, Massachusetts, Mississippi, New Hampshire, New Jersey, New York, North Carolina, Rhode Island, South Carolina, Texas, Virginia and Washington, DC, Fig. 3). Following our preregistered exclusion criteria^29^, 165 participants were screened out as they did not live in one of the target states, 12 participants were screened out after failing the attention check twice and 11 participants completed the survey in less than 4 min and were therefore excluded from the analysis. 168 participants did not complete the survey, and an additional 4 participants had missing answers for many of the forced response options, which suggests that they had connection issues that led to incomplete surveys. The final, complete sample of 4000 participants was reached within one week, namely between October 30th and November 1st, 2024. This period falls within the 2024 Atlantic Hurricane Season, but in this time frame there were no active storms threatening the continental United States. Participants were compensated for their time as well as additional bonus compensation for their performance on the quiz questions. We did not define or identify outliers. Data collection and analysis were not performed blind to the conditions of the experiments.

### Data preparation

Following the preregistration, we prepared the data on prior experience, by manually checking participants’ location of residence against past hurricane experience (identified through name, year, or both). For this, we used an online interactive hurricane tool (https://coast.noaa.gov/hurricanes). For those participants who listed a name (and optionally a year), we checked the respective hurricane path to assess whether it passed either close to the county of residence (approx. 100 km), or whether the (outer) rain bands of the hurricane could have affected this region. The latter was also checked through satellite imagery from https://zoom.earth/. For those participants who listed solely a year, we applied a search filter of approximately 100 km around their county of residence and checked whether there were any storm passages within this radius in the listed year. For those participants who listed a 2024 storm, we had to use either satellite imagery or Google Search, as the interactive hurricane tool did not contain data on 2024 on the date of investigation (January 29th, 2025). Based on these criteria, 203 respondents (9.5% of all 2136 who reported any past hurricane experience) were left out of the analysis for prior experience.

### Preregistered analysis: treatment tests

**Fig. 4:**
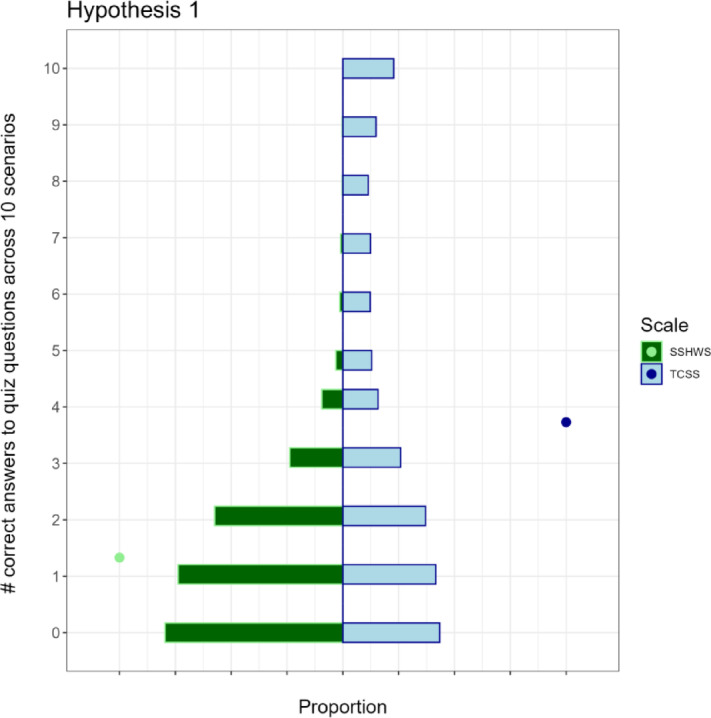
Distribution of correct quiz questions by treatment scale. Points represent the means for each scale.

*Hypothesis 1: Understanding the main hazard* To examine the impact of the TCSS and SSHWS scales on understanding of hurricane threats, we compared performance on the main hazard quiz across the four treatments. We hypothesized that participants exposed to TCSS warnings would be more accurate in identifying the main hazard than those exposed to SSHWS warnings. We tested this hypothesis with a two-tailed *t*-test comparing the number of scenarios (out of 10) in which the hazard question was answered correctly in the TCSS treatment and the SSHWS treatment. A significant difference between groups (with a higher mean in the TCSS) in quiz accuracy is interpreted as evidence that the TCSS leads to better understanding of the main hazards in hurricanes than the SSHWS scale. As hypothesized, we find that participants answer more quiz questions correctly in the TCSS treatment (3.7 questions on average) than in the SSHWS treatment (1.3 questions on average), (Welch 2-sample *t*-test, *t* =  − 29.971, df = 2617.75, *p*-value < 0.0001, 95% CI [− 2.56, − 2.24]) (Fig. 4).

**Fig. 5:**
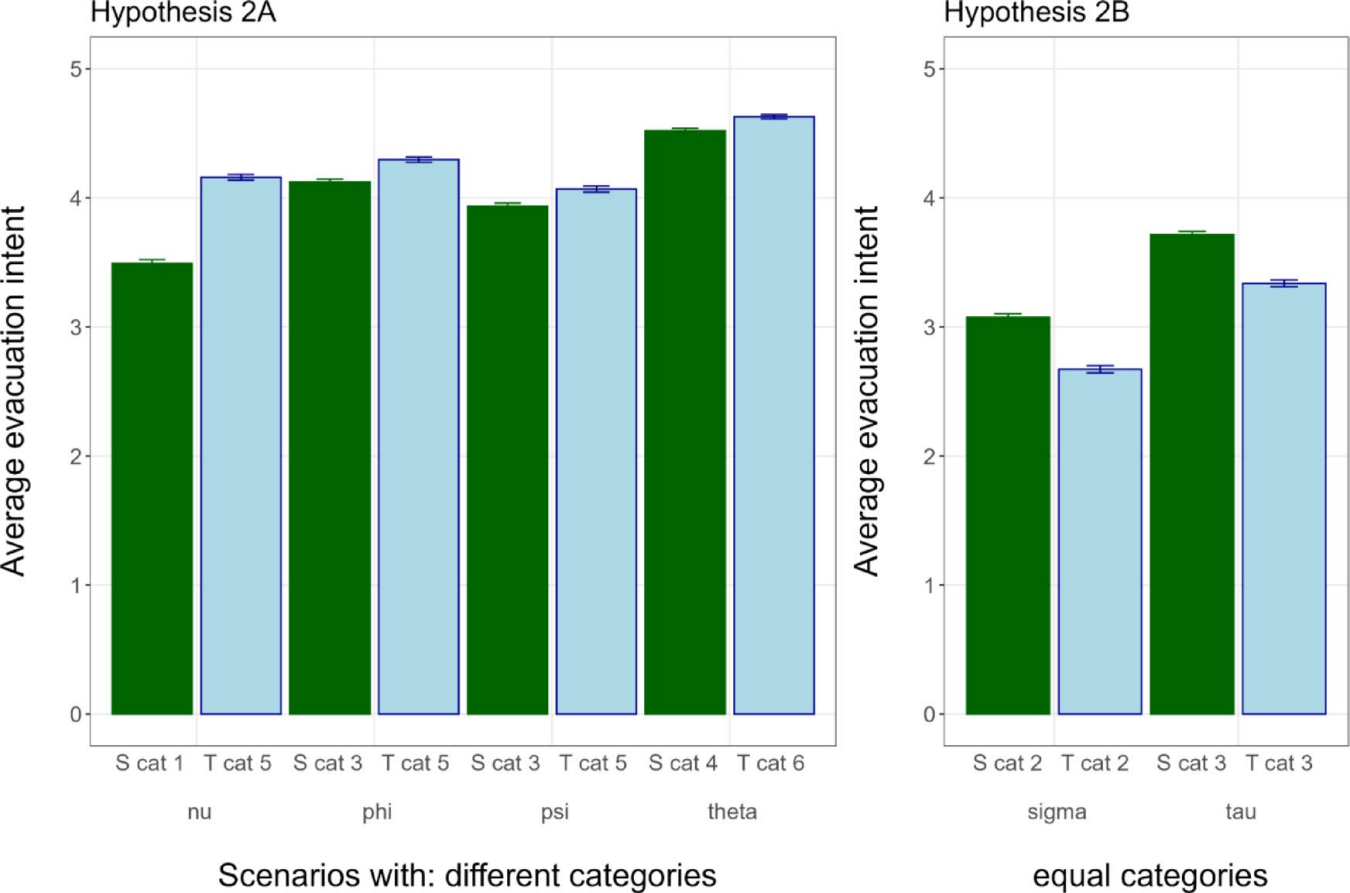
Average evacuation intent by scenario and treatment scale. Means and standard error bars are displayed. SSHWS (S) is represented in green bars, TCSS (T) in blue bars.

*Hypothesis 2: Evacuation intent* We compare evacuation intent for the four scenarios in which the TCSS category is 2 or more levels higher than the SSHWS category (H2a). We hypothesized that participants exposed to TCSS warnings would be more likely to evacuate in cases where the TCSS category is higher than the SSHWS category by 2 or more levels because these are the cases where non-wind hazards including rain and/or storm surge are more relevant and are not communicated by the SSHWS. We test this hypothesis with a two-tailed *t*-test comparing the average evacuation intent (in the 4 scenarios in which the TCSS category is 2 or more levels higher) across the TCSS and SSHWS treatments (Fig. 5).

We find that the average evacuation intent is indeed higher (in the 4 scenarios in which the TCSS category is 2 or more levels higher) in the TCSS treatment (mean = 4.3) than in the SSHWS treatment (mean = 4.1), (Welch 2-sample *t*-test, *t* =  − 5.654, df = 3981.23, *p*-value < 0.0001, 95% CI [− 0.23, − 0.11]). We also test for statistical equivalence in evacuation intent (TOST procedure)^30^ for the two scenarios with the same category across scales (H2b). In the scenarios with the same category across scales, we find a significantly lower evacuation intent in the TCSS scale (mean = 2.7) than in the SSHWS scale (mean = 3.1), (Welch 2-sample *t*-test, *t*(3994.6) = 10.184, *p*-value < 0.001, 95% CI [0.32, 0.48]). Further, the test for statistical equivalence using the TOST procedure (equivalence range − 0.15 to 0.15) was non-significant *t*(3994.6) = 6.39, *p* = 1, failing to reject the presence of a meaningful effect. Together, the significantly lower evacuation intent for TCSS compared to SSHWS and the non-significant statistical equivalence test provide evidence against hypothesis 2b.

**Fig. 6:**
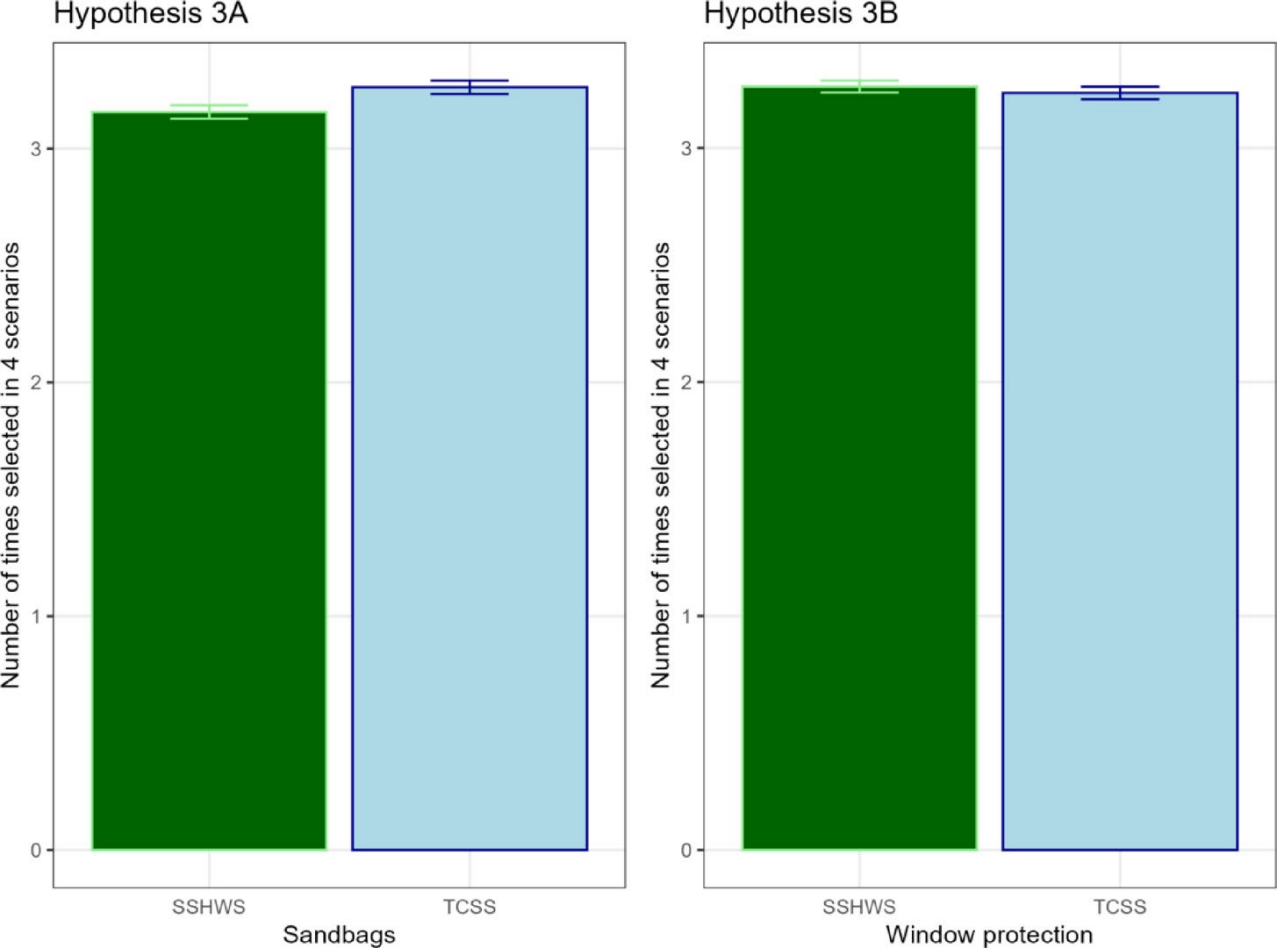
Adoption of precautionary measures by treatment scale. Means and standard error bars are displayed.

*Hypothesis 3: Measures taken* To examine the impact of the TCSS and SSHWS scales on measures taken, we compare adoption of wind-related and non-wind related measures for the same four scenarios as in H2a in which the TCSS category is 2 or more levels higher than the SSHWS category. We hypothesized that participants exposed to TCSS warnings will take more non-wind related measures (H3a), as measured by endorsement of using sandbags but that there would be no differences in wind-related measures (H3b), as measured by adoption of window protection (Fig. 6).

Indeed, we find a higher adoption of sandbag measures in the TCSS treatment group (mean = 3.24) than in the SSHWS treatment group (mean = 3.16), but the difference is not statistically significant at alpha = 0.005 (Welch 2-sample *t*-test, *t* =  − 2.595, df = 3997.89, *p*-value = 0.009, 95% CI [− 0.19, − 0.03]). However, a test for statistical equivalence with the TOST procedure (equivalence range − 0.15–0.15) was also not significant, *t*(3997.89) = 1.08, *p* = 0.14), which means that we cannot completely rule out the possibility of a meaningful effect. Therefore, the evidence in support of H3a is inconclusive as the difference in sandbag measures taken between SSHWS and TCSS is in the predicted direction, but neither statistically different at our preregistered alpha = 0.005 nor statistically equivalent. For the window protection measure, we test for statistical equivalence with the TOST procedure (equivalence range − 0.15–0.15). The equivalence test was significant *t*(3993.7) =  − 3.31, *p* < 0.001 and the null hypothesis test was non-significant, *t*(3993.7) = 0.734, *p* = 0.46. The significant equivalence test rejects the presence of a meaningful effect of TCSS versus SSHWS scale on window protection, evidence in support of H3b.

### Preregistered analysis: panel regressions

*Hypothesis 1: Understanding the main hazard* We further test our hypothesis in a linear probability panel regression including all scenarios, predicting quiz score by treatment dummy, controlling for gender, age, home ownership and previous hurricane experience. We cluster standard errors at the individual level to account for the repeated-measures design in which each individual has gone through all 10 scenarios. We used linear probability models because interactions in non-linear models are not straightforward to interpret, and their magnitude and sign may be very sensitive to different covariates^31^. Table 2 shows the results (see Supplementary Table S3 for robustness to logit and probit specifications).

**Table 2:** Linear probability panel regression of quiz questions.

	Dependent variable: # correct quiz questions
Constant	0.238***
	(0.014)
TCSS treatment (ref = SSHWS)	0.243***
	(0.008)
Gender female (ref = male)	0.007
	(0.008)
Age	− 0.003***
	(0.0003)
Homeowner (1 = yes)	0.001
	(0.009)
Hurricane experience (1 = yes)	0.020
	(0.009)
Evacuation experience (1 = yes)	− 0.037
	(0.015)
Adjusted R^2^	0.0254
N	3796

Robust standard errors in parentheses (*p < 0.005; **p < 0.001; ***p < 0.0005).

We find a positive coefficient for the TCSS treatment dummy (SSHWS was used as the reference category) in a linear probability panel regression including all scenarios, predicting quiz score and controlling for gender, age, homeownership and previous hurricane experience (β = 0.243, *p*-value < 0.0001, 95% CI [0.23, 0.26]). This means that the TCSS treatment led to more correct identifications of the main hazard across scenarios.

*Hypothesis 2: evacuation intent* Furthermore, as preregistered, we tested our hypotheses in panel regressions in Table 3. We include all scenarios, predicting our dependent variables evacuation intent, sandbag measures, and window measures taken, interacting with the wind, rainfall and storm surge category of the respective scenario, controlling for gender, age, homeownership and previous hurricane experience.

**Table 3:** Panel regressions of evacuation intent and precautionary measures.

	Dependent variable					
	Evacuation intent (op)		Sandbags (lpm)		Window protection (lpm)	
	(1)	(2)	(3)	(4)	(5)	(6)
Constant	0.660***	1.827***	0.472***	0.715***	0.631***	0.807***
	(0.115)	(0.036)	(0.020)	(0.007)	(0.017)	(0.006)
Treatment TCSS (ref = SSHWS)	− 0.506***	− 0.038	− 0.084***	− 0.015	− 0.066***	− 0.033***
	(0.077)	(0.057)	(0.015)	(0.010)	(0.013)	(0.009)
Wind category	0.447***		0.031***		0.070***	
	(0.008)		(0.002)		(0.003)	
Rainfall category	0.154***		0.035***		0.0003	
	(0.005)		(0.002)		(0.001)	
Storm surge category	− 0.022***		0.012***		− 0.017***	
	(0.005)		(0.002)		(0.002)	
TCSS × wind category	− 0.105***		− 0.008		− 0.003	
	(0.011)		(0.003)		(0.004)	
TCSS × rainfall category	0.170***		0.021***		0.009***	
	(0.007)		(0.002)		(0.002)	
TCSS × storm surge category	0.100***		0.011***		0.006	
	(0.008)		(0.002)		(0.002)	
Log likelihood	− 44,903.86	− 54,599.62	− 12,045.05	− 15,039.85	− 9133.02	− 11,253.82
Adjusted R^2^			0.1009	0.00005	0.0663	0.00034
N	3796	4000	3796	4000	3796	4000

Robust standard errors in parentheses (alpha level Bonferroni corrected: *p < 0.0025; **p < 0.0005; ***p < 0.00025). Covariates suppressed for brevity: gender, age, homeownership, previous evacuation experience.

A significant positive interaction of the TCSS treatment dummy with the rainfall hazard and/or the storm surge hazard is interpreted as evidence that the TCSS leads to higher evacuation intent for non-wind hazards than the SSHWS, controlling for various covariates. For this test we applied a Bonferroni correction to account for multiple hypothesis testing, leading to alpha = 0.0025.

Indeed, we find that TCSS leads to higher evacuation intent for rainfall hazards: β = 0.17,* p*-value < 0.0001, 95% CI [0.156, 0.183] as well as for storm surge: β = 0.10, *p*-value < 0.0001, 95% CI [0.084, 0.115].

*Hypothesis 3: precautionary measures* A significant positive coefficient of the interaction between the TCSS treatment and storm surge hazard or rainfall hazard in sandbags as precautionary measures is interpreted as evidence that the TCSS leads to a better response in terms of precautionary measures, controlling for various covariates. Again, in support of H3a, we find a significant positive interaction between TCSS and rainfall and storm surge on endorsement of sandbag use (TCSS × rainfall β = 0.021, *p*-value < 0.0001, 95% CI [0.016, 0.026]), (TCSS × storm surge β = 0.011, *p*-value < 0.0001, 95% CI [0.007, 0.016]). However, we find a smaller magnitude, but still significant interaction between TCSS and rainfall on the wind-related measure of window protection (TCSS × rainfall β = 0.009, *p*-value < 0.0001, 95% CI [0.006, 0.013]) which goes against our predicted lack of difference in H3b, but the interaction between TCSS and storm surge is not significant at alpha = 0.0025 (TCSS × storm surge β = 0.006, *p*-value = 0.008, 95% CI [0.002, 0.010]) offering mixed support for H3b. See supplementary Table S4 for robustness of the results to logit and probit specifications.

### Exploratory analysis

In addition to the hypotheses specified above, we explored the impact of various (mostly confounding) variables on the intent of taking action (evacuation and precautionary measures). Specifically, these include:

- Text only versus text and a graphic. We expected H1-3 to hold regardless of presentation format, but we test whether graphics impact any outcomes and whether there is any interaction between scale and graphics on outcomes.
- Prior hurricane experience. We expected this to have a positive effect on participant’s intent on taking action.
- Near-miss event experience. We expected this to have a negative effect on participant’s intent on taking action.
- Identification of the main hazard. We expected this to be a mediator, with a positive effect on participant’s intent on taking action appropriate for that hazard.
- Risk perception. We expected this to be a mediator, through which participants take action.
- Measures of information-gathering: clicking for additional information, time spent on additional information, and overall response time on page. We tested whether these measures mediate accuracy on quiz questions.

The panel regression shows that the treatment: text only vs text and graphics, did not influence the results on evacuation intent significantly (Table 4). This indicates that results can be pooled as is done in our analysis (for further tests, including interactions, see Figs. S3, S4, S6 and Table S2 in Supplementary information). Most importantly, the regressions show that identifying the main hazard corresponds with significantly higher intent for the proper measure: identification of wind as main hazard results in higher window protection (β = 0.135, *p*-value < 0.0001, 95% CI [0.12, 0.15]) and identification of rainfall or storm surge as main hazard result in higher intent to put sandbags in place (β = 0.154, *p*-value < 0.0001, 95% CI [0.14, 0.17] and β = 0.118, *p*-value < 0.0001, 95% CI [0.11, 0.13] respectively). Overall, it appears that risk perception has a very strong influence on intent to take specific measures. Contrastingly, we see no indication that prior (near miss) experience with hurricanes influences the intent to take specific measures. We find that time on page and clicking for more information negatively affect the amount of quiz questions answered correctly. In the supplement, we also test for differences between states and do not find any significant differences at our alpha = 0.005 (Supplementary Table S5).

## Discussion

Whilst we did find evidence for increased evacuation intent under the TCSS when it has a higher category than SSHWS (H2a), we surprisingly found a lower evacuation intent with TCSS in cases where the category number is equal (where we hypothesised that this would be equal), meaning we need to reject H2b. One possible explanation could be related to potential overestimation of the hazard by respondents when they need to interpret feet of storm surge, inches of rainfall and miles/hour of wind speed. In both SSHWS and TCSS warnings, we provide the numbers, but in the TCSS warnings these numbers are accompanied with a category classification. It may be that, for example, an 8 feet storm surge or 120 miles/hour wind speed is interpreted as quite hazardous (resulting in more evacuation), but when classified as ‘only’ category 2 or 3 people feel less in danger (resulting in less evacuation). Future interviews or focus groups could investigate if this overestimation, when participants are interpreting data by themselves, is indeed happening. Another potential factor could be people’s sensitivity towards hurricane categories and their preparedness to evacuate or take precautionary measures^32^. Collins et al.^32^ surveyed hurricane decision making for Hurricanes Laura and Sally. Upon making landfall, Hurricane Sally was a Category-2 hurricane that brought around 30 inches (750 mm) of rainfall upon making landfall. Various participants of the authors’ surveys mentioned that they typically only evacuate for hurricanes of Category 3 and up, illustrating people’s sensitivity towards a hurricane category and their evacuation intent. Noteworthy is that many respondents said they were taken by surprise by Sally’s torrential and impactful rainfall, indicating that they felt the SSHWS Category ranking had misled them in believing the storm would not have been so hazardous. In a sensitivity analysis of evacuation intent by TCSS category, TCSS compared to SSHWS leads to lower evacuation intent for categories 3 and lower, and higher evacuation intent for categories 5 and 6 (Supplementary Fig. S7). This provides suggestive evidence that TCSS may lead to more evacuations for the most dangerous storms, and reduced evacuations for the least dangerous. However, the reduced evacuation for category 3 storms should be further investigated as this is often a key threshold for evacuation.

With respect to triggering precautionary measures, we find that TCSS or SSHWS has no different impact on choosing window protection (confirming H3b; Fig. 6). However, whilst we do see a higher adoption of sandbags with the TCSS treatment group, the difference is small and not significant at our stated 0.005 level (*p*-value = 0.009), making it inconclusive to confirm H3a. In addition, the regression (Table 3) shows that the interaction of TCSS with rainfall and storm surge categories leads, as hypothesized in H3a, to significantly more sandbag usage (the appropriate precautionary measure against flooding hazard). Indeed, a recent focus group study on different hurricane scales found that “many participants saw the TCSS as an improvement, valuing its separate ratings for wind, surge, and rain” suggesting that being able to identify the main hazard is useful for choosing appropriate precautionary measures and actions^33^.

In the exploratory analysis (Table 4), a few surprising results were observed. First, we did not find any evidence that prior hurricane experience correlated with precautionary measures of sandbags and window protection. We did find a significant effect of prior experience on evacuation intent, but the sign of this was negative, indicating lower evacuation intent among respondents with prior hurricane experience. This can be due to either people’s evacuation during a previous hurricane being perceived as not necessary in retrospect (the so-called “crying wolf” effect), or that during a previous hurricane they did not evacuate and did not get into trouble. In both cases, someone would have lower evacuation intentions in the future. The lack of correlation with implementing sandbags and window protection is more difficult to explain. It could be that these measures are more specific, and therefore less frequently taken (during previous hurricanes), resulting in less experience with them and less influence on behavioural intentions. In other words, prior hurricane experience does not necessarily translate to prior experience with these precautionary measures.

**Table 4:** Panel regressions exploring the effect of confounding variables on the intent of taking action.

	Dependent variable			
	Quiz correct (lpm)	Evacuation intent (op)	Sandbags (lpm)	Window protection (lpm)
Constant	0.140***	− 3.923***	0.008	0.261***
	(0.005)	(0.067)	(0.016)	(0.015)
Treatment text (ref = graphic)	0.021	− 0.030	− 0.008	− 0.008
	(0.008)	(0.029)	(0.009)	(0.008)
Treatment scale (ref = SSHWS)	0.238***	0.031	− 0.017	− 0.003
	(0.008)	(0.029)	(0.009)	(0.008)
Prior hurricane experience (1 = yes)		− 0.209***	0.011	0.006
		(0.033)	(0.010)	(0.009)
Near miss experience (1 = yes)		0.010	− 0.011	− 0.024
		(0.039)	(0.014)	(0.013)
Identified main hazard as wind		0.074***	0.012	0.135***
		(0.018)	(0.006)	(0.006)
Identified main hazard as rainfall		0.147***	0.154***	− 0.022***
		(0.017)	(0.006)	(0.005)
Identified main hazard as storm surge		0.365***	0.118***	0.008
		(0.018)	(0.006)	(0.005)
Risk perception		1.783***	0.144***	0.115***
		(0.017)	(0.003)	(0.003)
Clicked for more information	− 0.048***	0.047	0.011	0.024***
	(0.007)	(0.029)	(0.007)	(0.006)
Time on page	− 0.0002***	0.0002	− 0.00001	− 0.00002
	(0.00003)	(0.001)	(0.00003)	(0.00002)
Log likelihood	− 14,739.22	− 31,979.62	− 9751.26	− 7808.67
N	4000	3797	3797	3797

Robust standard errors in parentheses (*p < 0.005; **p < 0.001; ***p < 0.0005).

Second, in the quiz results (Fig. 4) we find, as expected in H1, the respondents in the TCSS treatment more frequently correctly identify the main hazard(s). However, we also find a surprisingly large number of respondents (both SSHWS and TCSS) with very few (zero or one) correct answers. This may be related to the design of the forecast with multiple hazards (sometimes of equal category) and that we used only one correct answer. In the TCSS treatment, we do find many respondents with ten out of ten questions correct, which gives confidence that the TCSS provides information to the reader as intended. Further, we tested alternative, more flexible criteria for the main hazards and found that quiz performance doubled from an average of 2.5 to 5 correct questions. Nevertheless, the TCSS still significantly outperformed the SSHWS (Supplementary Table S1, Fig. S1).

Lastly, we find that the time spent on the page and clicking for more information actually leads to fewer correct quiz results. Intuitively, one might expect that people who seek out more information and spend more time answering a question will have better results (as opposed to ones who click through). The opposite seems to be the case, however. This suggests that clicking for more information and spending a longer time on the page relates more to respondents who are not sure about the answer looking more and longer to try and find an answer, a phenomenon also known as milling^9^. Respondents who understand the information well are fast to identify the correct answer and do not need to click further (as all the information needed for a correct answer can be found on the opening page).

## Conclusion

In this paper we compared the current Saffir-Simpson Hurricane Wind Scale (SSHWS; solely based on wind intensity) with the newly developed Tropical Cyclone Severity Scale (TCSS; based on rainfall, storm surge and wind intensities) to determine if a change in scale improves understanding of the risks posed by a tropical cyclone, and how it changes decisions related to evacuating and implementing measures. Our results show that the TCSS results in a substantially better identification of the main hazard (confirming H1). In turn, correct identification of the main hazard increases the intent to take relevant precautionary measures (i.e. sandbags for rainfall and storm surge hazards, and window protection for wind hazards). Overall, we see that respondents exposed to the TCSS warnings have higher intent to select relevant measures related to non-wind hazards as opposed to respondents exposed to SSHWS, though this is not significant at our 0.005 level (making H3a inconclusive). We do see, as hypothesized in H3b, that the intention to implement window protection does not differ between SSHWS and TCSS (which have the same category numbers for wind).

With respect to evacuation, we find that the TCSS leads to significantly higher evacuation intent as opposed to SSHWS in cases where the TCSS is at least two points higher (due to rainfall or storm surge being the main hazard), confirming H2a. Interestingly, we see that the evacuation intent for storms that have the same category between TCSS and SSHWS (thus where wind is the main hazard), shows a lower evacuation intent for TCSS (against H2b which hypothesized no difference). This may be related to respondents’ inclinations to evacuate for higher category storms (4 and 5), and not for lower ones (1, 2, 3) given that our results stem from a category 2 and 3 storm (Sigma and Tau). This would imply that some respondents overestimate the danger of rainfall or storm surge if they have to interpret its danger themselves (with the SSHWS) from given inches and feet; whereas with the TCSS this is done for them by providing a (low) rainfall and storm surge categorization.

Overall, our results demonstrate that people make better informed and more appropriate decisions with the TCSS as opposed to the current SSHWS. Institutions in charge of operational forecasting and public communication and warning should thus strongly consider transitioning towards a scale that conveys information on all primary hazards associated with a landfalling tropical cyclone (such as the TCSS). This means that next to wind speed, also rainfall and storm surges have to be forecasted in an operational setting, to be integrated in the communication or warning. In the United States, information on rainfall or storm surges already often accompany hurricane warnings, implying this is not necessarily a barrier for implementation. Moreover, the TCSS allows for spatial differentiation in category and nature of the main hazard for different areas, enabling more precise communication about which specific hazard people should prepare for.

## Supplementary Information

Supplementary Information.

## Data Availability

All data and materials are available on the Open Science Framework at (https://osf.io/d4pje/).

## Code availability

All analysis code is available on the Open Science Framework at (https://osf.io/d4pje/).
