# Supplementary material for: An experimental test of risk perceptions under a new hurricane classification system
Source: Sci Rep. 2025 Aug 19;15:30320. doi: 10.1038/s41598-025-14170-1 (PMC12365295; doi:10.1038/s41598-025-14170-1)
Supplement: Supplementary file 1 — Supplementary Information. [file 41598_2025_14170_MOESM1_ESM.pdf]

Stage 2 Supplementary Information

Main hazard identification robustness

We tested the robustness of the treatments in identifying the main hazards of the scenarios by using additional, more relaxed criteria. For the relaxed criteria, we allowed any hazard with a category 3 or higher to count as a main hazard. This is because hazards with values over 3 can be considered more serious, both in qualitative research[1] and because the SSHWS category 3 and higher is classified as a major hurricane whereas lower categories are not. For these reasons, it may be advisable to recognize hazards with a category 3 and higher as dangerous. In Table S1, all acceptable answers for each scenario are displayed with the additional relaxed criteria bolded. Note that this does not apply to hurricane Theta in which all hazards are over 3 because we did not have an "all hazards" option, so only rainfall and storm surge can be correct.

Table S1. Overview of scenarios with relaxed criteria for main hazard.

Name	Wind (mph)	Surge (feet)	Rain (inches)	Category SSHWS	Category TCSS	Historical example	Main hazard
Chi	120 (3)	8 (2)	24 (4)	3	4	Irma (2017)	Rainfall or **Rainfall/ Wind**
Lambda	70 (0)	4 (1)	8 (1)	0	1	Gordon (2018)	Rainfall/Storm surge
Omega	100 (2)	4 (1)	20 (3)	2	3	Alex (2010)	Rainfall
Nu	85 (1)	10 (4)	31 (5)	1	5	Florence (2018)	Rainfall or **Rainfall/Storm** surge
Rho	145 (4)	20 (5)	8 (1)	4	5	Emily (2005)	Storm surge or **Storm surge/Wind**
Sigma	100 (2)	4 (1)	8 (1)	2	2	Bertha (1996)	Wind
Tau	120 (3)	8 (2)	8 (1)	3	3	Fran (1996)	Wind
Phi	120 (3)	20 (5)	12 (2)	3	5	Katrina (2005)	Storm surge or **Storm surge/Wind**
Theta	145 (4)	20 (5)	31 (5)	4	6	Wilma (2005)	Rainfall/Storm surge
Psi	120 (3)	4 (1)	31 (5)	3	5	Sally (2020)	Rainfall or **Rainfall/Wind**

Notes: We list values in U.S. customary units, which is how they were stated in the experiment, since our target group is familiar with this unit system. Wind, surge, and rain columns indicate values in mph, feet and inches respectively, followed by the individual TCSS category in parentheses. Note that Hurricane Theta was slightly different in the pilot (wind speed 154 mph / category 5). The relaxed main hazards include any hazard with a category of 3 or higher in the TCSS scale instead of only that with the highest value with the exception of hurricane Theta because there was no "all hazards" option. The additional correct options are bolded.

Comparing the different criteria, average quiz performance is 2.5 questions correct with the strict criteria and this increases to an average of 5 questions correct with the relaxed criteria. This suggests that participants may indeed perceive hazards of category 3 and higher as main hazards even if they are not the highest category threat as it allows them to weigh all potential threats. Comparing the treatments within the relaxed criteria, participants in the TCSS treatment (5.9 questions on average) significantly outperform those in the SSHWS treatment (4.0 questions on average), (Welch 2-sample t-test, $t = -25.65$, $df = 3353.2$, $p\text{-value} < 0.0001$, 95% CI [-2.06, -1.77]). The distributions of correct answers for each treatment are plotted in Figure S1.

[1] Collins, J., Polen, A., Dunn, E., Jernigan, I., McSweeney, K., Welford, M., ... & Zhu, Y. J. (2022). Hurricanes Laura and Sally: A case study of evacuation decision-making in the age of COVID-19. Weather, Climate, and Society, 14(4), 1231-1245.

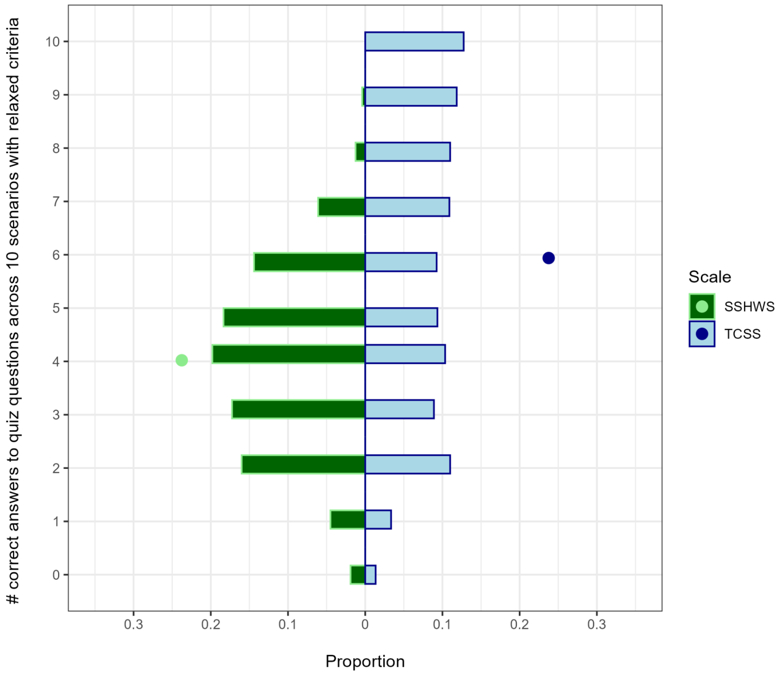

Figure S1: Quiz performance with relaxed criteria: the proportion of correct identification of the main hazard split by scale, TCSS versus SSHWS, allowing for multiple hazards to be the main hazard if both have a category over 3 in the TCSS scale. Points represent the means for each scale.

Main hazard all scenarios

For all scenarios, the TCSS outperforms the SSHWS in the percentage of participants getting the quiz question correct, but this is more pronounced for hurricanes where wind is not the main hazard (Figure S2).

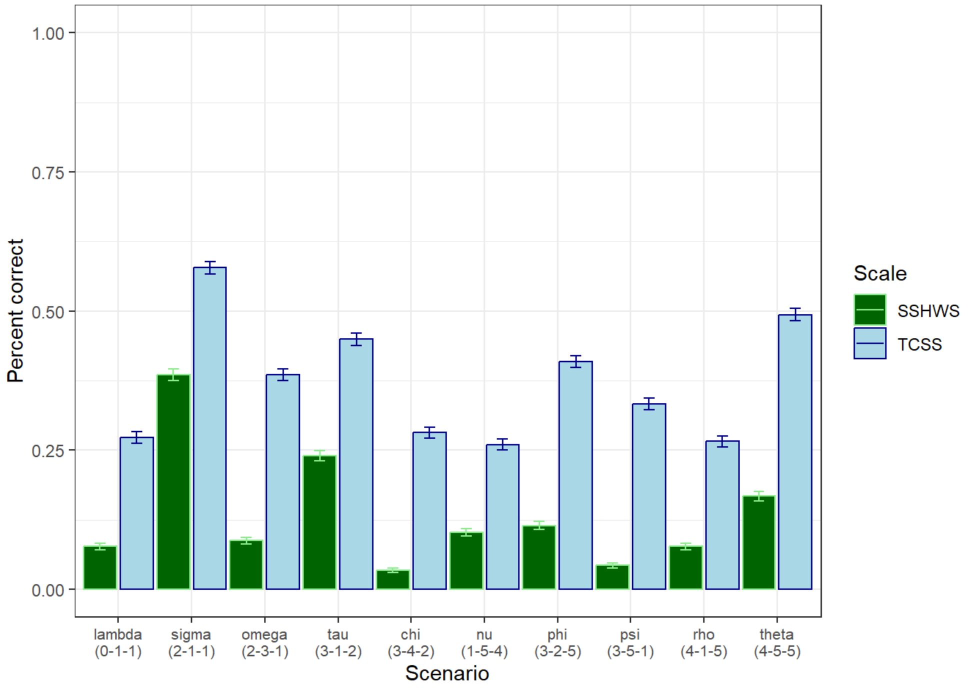

Figure S2: Quiz performance split by scenario: the proportion of correct identification of the main hazard split by scale, TCSS versus SSHWS, for each specific hurricane scenario. Error bars represent standard errors.

Main results split by scale and presentation form (text vs. graphics)

We expected H1 to hold regardless of presentation format, but we tested whether graphics had any impact on the quiz accuracy. Participants did answer slightly more quiz questions correctly in the text treatment (2.6 questions on average) than in the graphics treatment (2.4 questions on average), but this is not significant at our preregistered alpha = 0.005 (Welch 2-sample *t*-test, t = -2.36, df = 3995.49, p-value = 0.018, 95% CI [-0.38, -0.04]). In all, this small difference does not change the main finding that the TCSS communicates the main hazard better than SSHWS regardless of presentation format.

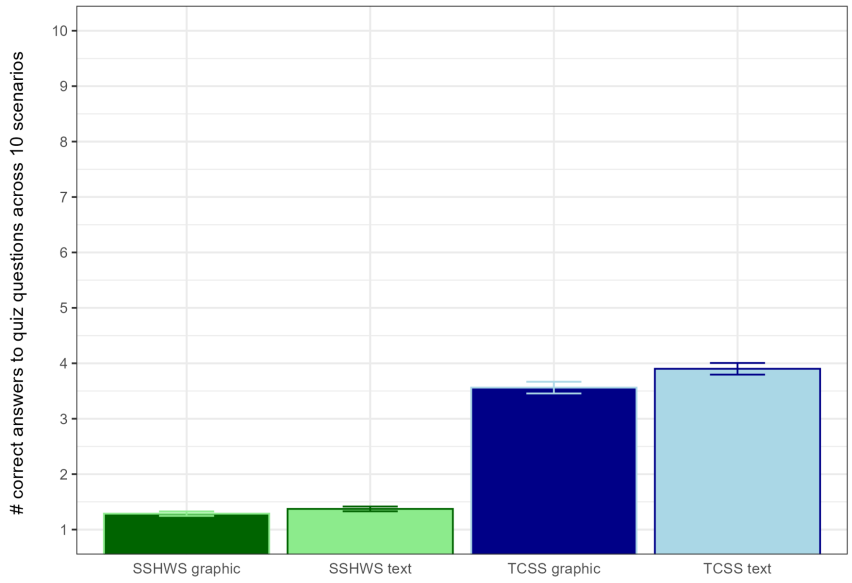

Figure S3: Quiz performance split by scale and format (text versus graphic): the number of correct quiz questions out of a maximum of 10 split by scale (TCSS vs SSHWS) and format (text or graphic). Error bars represent standard errors..

We also expected H2 to hold regardless of presentation format, and we tested whether graphics had any impact on the evacuation intent. We find no significant difference in the average evacuation intent in the 4 scenarios in which the TCSS category is 2 or more levels higher between the text treatment (mean = 4.2) and the graphic treatment (mean = 4.2), (Welch 2-sample t -test, t = 1.856, df = 3996.38, p-value = 0.064, 95% CI [0.00, 0.12]). Further, in scenarios with the same category across scales, we again find similar average evacuation intent in the text treatment (mean = 2.9) and the graphic treatment (mean = 2.9), (Welch 2-sample *t*-test, t = -0.03, df = 3996.49, p-value = 0.976, 95% CI [-0.08, 0.08]). Together, this suggests that the presentation format did not influence our findings in H2.

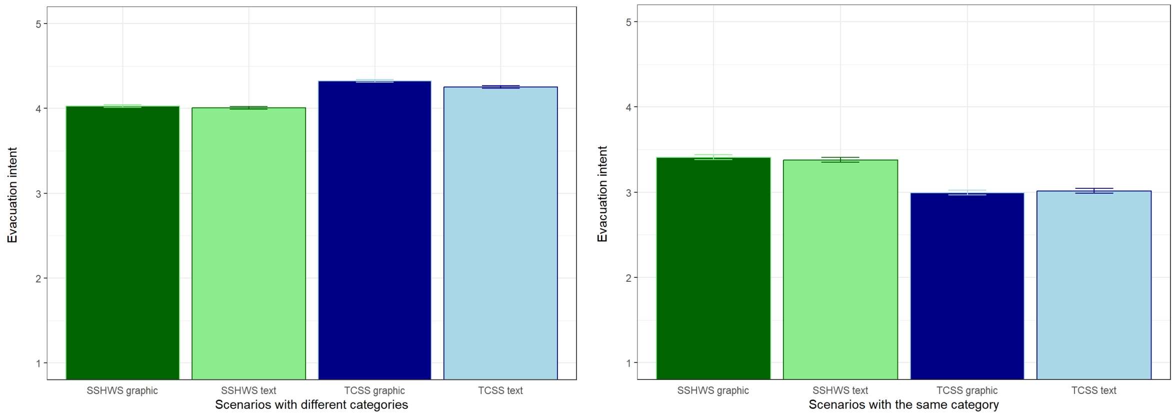

Figure S4: Evacuation intent split by scale and format (text versus graphic): Evacuation intent in A) scenarios where the TCSS gives a higher category than SSHWS or B) the same category as SSHWS split by scale and format. Error bars represent standard errors.

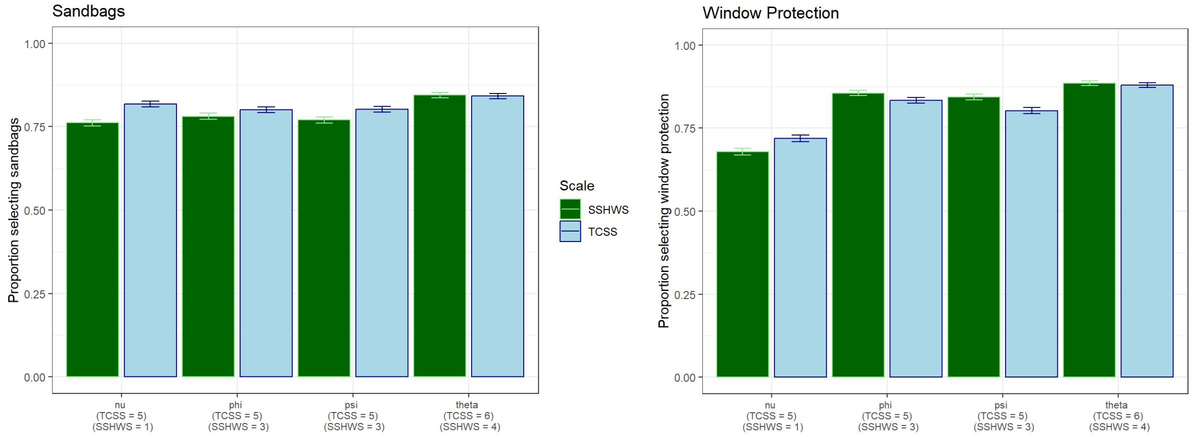

Figure S5: Measures taken split by scenario: Endorsement of A) Sandbag use or B) window protection across all scenarios where the TCSS gives a higher category (by 2 or more) than SSHWS split by scale. Error bars represent standard errors.

As with H1 and H2, we expected H3 to hold regardless of presentation format, and we tested whether graphics versus text had any impact on the endorsement of sandbag use or window protection. We do not find significant differences between formats for the number of times sandbag measures were endorsed in the text treatment (mean = 3.2) versus the graphics treatment (mean = 3.2), (Welch 2-sample *t*-test, $t = 0.848$, df = 3996.15, *p*-value = 0.397, 95% CI [-0.05, 0.11]). We also do not find any differences in the number of times window protection measures were endorsed in the text treatment (mean = 3.2) compared to the graphic treatment (mean = 3.3), (Welch 2-sample *t* -test, $t= 0.957$, df = 3997.83, *p*-value = 0.339, 95% CI [-0.04, 0.11]).

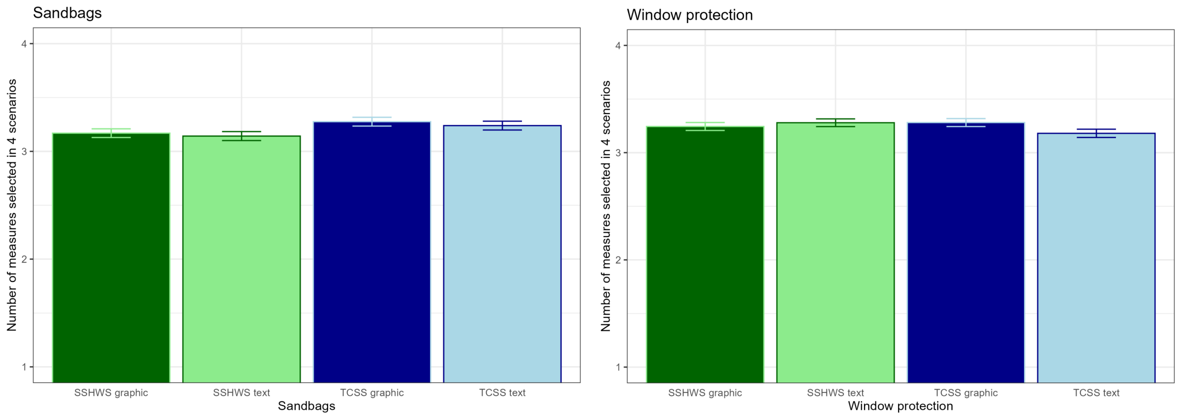

Figure S6: Measures taken split by scale and format (text versus graphic): Endorsement of A) Sandbag use or B) Window protection in the 4 scenarios where the TCSS gives a higher category than SSHWS split by scale and format. Error bars represent standard errors.

In Table S2 below, we tested whether graphics had any impact on the outcomes and whether there is any interaction between scale type and format on outcome. In linear regressions, we do not find any main effect of treatment format or any significant interactions between treatment scale and format at our preregistered alpha = 0.005 on any of our outcome variables.

Table S2. Interactions between treatment scale and format on different outcomes

	Dependent variable:				
	Quiz correct	Evacuation diff. cat.	Evacuation same cat.	Sandbags	Window protection
Constant	1.287***	4.141***	3.102***	3.171***	3.246***
	(0.080)	(0.030)	(0.040)	(0.041)	(0.037)
treatment TCSS (ref = SSHWS)	2.274***	0.193***	-0.458***	0.111	0.041
	(0.113)	(0.043)	(0.056)	(0.058)	(0.052)
treatment text (ref = graphic)	0.086	-0.035	-0.055	-0.029	0.033
	(0.113)	(0.043)	(0.056)	(0.058)	(0.052)
treatment TCSS x treatment text	0.254	-0.042	0.111	-0.010	-0.137
	(0.160)	(0.061)	(0.079)	(0.082)	(0.074)
Observations	4,000	4,000	3,999	4,000	4,000
R^2	0.185	0.009	0.026	0.002	0.001
Adjusted R^2	0.185	0.008	0.025	0.001	0.0005
Residual Std. Error	2.531 (df = 3996)	0.961 (df = 3996)	1.249 (df = 3995)	1.289 (df = 3996)	1.173 (df = 3996)
F Statistic	302.954*** (df = 3; 3996)	11.969*** (df = 3; 3996)	35.235*** (df = 3; 3995)	2.488 (df = 3; 3996)	1.614 (df = 3; 3996)

Notes: Linear regressions. (*p<0.005; **p<0.0025; ***p<0.0005)

Evacuation sensitivity to category by scale

In an exploratory analysis of sensitivity to TCSS category, participants in the TCSS treatment show evacuation intent more in line with the TCSS scale, whereas those in the SSHWS indicate higher evacuation intent at lower categories (<4) and lower evacuation intent at higher categories (>4) with a crossover point around category 4.

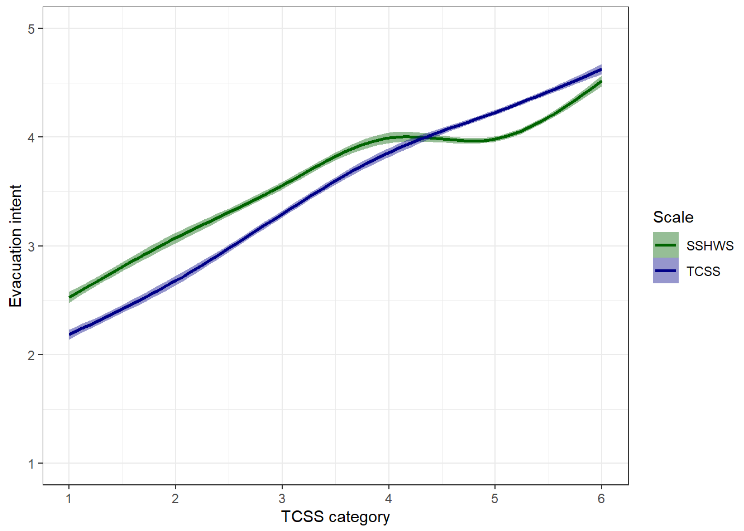

Figure S7: Evacuation intent by TCSS category split by treatment scale: the figure shows how evacuation intent changes with TCSS category for the SSHWS and TCSS treatments across all 10 scenarios. Lines represent smoothed raw data with the shaded band representing the 95% confidence interval.

Robustness of regression results to logit and probit specifications

Tables S3 and S4 replicate Tables 2 and 3 from the main analyses but with logit and probit specifications instead of linear probability regressions.

Table S3. Table 2 in logit and probit specification

	Dependent variable:	
	correct questions	
	logit	probit
Constant	-1.280***	-0.768***
	(0.044)	(0.026)
TCSS treatment (ref = SSHWS)	1.381***	0.800***
	(0.026)	(0.015)
Gender female (ref = male)	0.040	0.019
	(0.025)	(0.015)
Age	-0.017***	-0.010***
	(0.001)	(0.001)
Homeowner (1 = yes)	0.010	0.006
	(0.026)	(0.015)
Hurricane experience (1 = yes)	0.117***	0.063***
	(0.026)	(0.015)
Evacuation experience (1 = yes)	-0.227***	-0.125***
	(0.050)	(0.029)

Notes: Robust standard errors in parentheses (**p<0.01; **p<0.005;* ***p<0.001). Covariates suppressed for brevity: gender, age, homeownership, previous evacuation experience.

Table S4. Table 3 in logit and probit specifications

	Dependent variable:			
	Sandbags (logit)	Sandbags (probit)	Window protection (logit)	Window protection (probit)
Constant	-0.390***	-0.218***	0.186*	0.133***
	(0.057)	(0.034)	(0.061)	(0.036)
treatment TCSS (ref = SSHWS)	-0.400***	-0.237***	-0.311***	-0.188***
	(0.060)	(0.036)	(0.062)	(0.037)
Wind category	0.173***	0.106***	0.468***	0.271***
	(0.017)	(0.010)	(0.018)	(0.010)
Rainfall category	0.157***	0.093***	-0.024	-0.013
	(0.010)	(0.006)	(0.012)	(0.007)
Storm surge category	0.114***	0.067***	-0.019	-0.014
	(0.011)	(0.007)	(0.013)	(0.007)
TCSS × wind category	-0.043	-0.024	-0.083*	-0.044*
	(0.025)	(0.014)	(0.024)	(0.014)
TCSS × rainfall category	0.104***	0.059***	0.026	0.015
	(0.015)	(0.008)	(0.016)	(0.009)
TCSS × storm surge category	0.066***	0.037***	0.093***	0.053***
	(0.016)	(0.010)	(0.018)	(0.010)

Notes: Robust standard errors in parentheses (*p<0.005; **p<0.0025; ***p<0.0005). Covariates suppressed for brevity: gender, age, homeownership, previous evacuation experience.

Robustness of analysis to differences between states

There were no significant differences between states in any of our main outcome metrics at our alpha = 0.005 significance level and none of the results change when accounting for state (Table S5).

Table S5. Accounting for states in different outcomes

	Dependent variable:				
	Quiz correct	Evacuation diff. cat.	Evacuation same cat.	Sandbags	Window protection
Constant	1.086***	4.216***	3.100***	3.136***	3.194***
	(0.229)	(0.087)	(0.113)	(0.117)	(0.106)
treatment TCSS (ref = SSHWS)	2.273***	0.189***	-0.466***	0.112	0.040
	(0.113)	(0.043)	(0.056)	(0.058)	(0.052)
treatment text (ref = graphic)	0.076	-0.030	-0.060	-0.014	0.039
	(0.113)	(0.043)	(0.056)	(0.058)	(0.053)
treatment TCSS x	0.259	-0.048	0.120	-0.022	-0.143
treatment text	(0.160)	(0.061)	(0.079)	(0.082)	(0.074)
Connecticut	0.185	-0.243	-0.243	-0.074	-0.028
	(0.347)	(0.132)	(0.171)	(0.177)	(0.161)
Delaware	0.092	-0.540	-0.317	0.098	0.065
	(0.542)	(0.206)	(0.267)	(0.276)	(0.251)
District of Columbia	1.623	0.022	-0.035	0.181	0.298
	(0.620)	(0.236)	(0.306)	(0.316)	(0.288)
Florida	0.045	-0.087	-0.182	0.211	0.119
	(0.241)	(0.092)	(0.119)	(0.123)	(0.112)
Georgia	-0.089	-0.025	0.117	0.018	0.043
	(0.255)	(0.097)	(0.126)	(0.130)	(0.118)
Louisiana	0.075	0.034	-0.171	-0.090	-0.084
	(0.327)	(0.124)	(0.161)	(0.166)	(0.151)
Maine	0.964	0.155	0.167	-0.117	0.257
	(0.518)	(0.197)	(0.255)	(0.264)	(0.240)
Maryland	0.309	-0.051	-0.021	0.024	-0.008
	(0.297)	(0.113)	(0.147)	(0.152)	(0.138)
Massachusetts	0.803	-0.118	-0.069	0.202	0.094
	(0.304)	(0.115)	(0.150)	(0.155)	(0.141)
Mississippi	0.076	-0.034	-0.233	0.073	0.213
	(0.366)	(0.139)	(0.181)	(0.187)	(0.170)
New Hampshire	0.781	-0.048	0.207	-0.003	-0.176
	(0.480)	(0.182)	(0.237)	(0.245)	(0.223)
New Jersey	0.069	-0.098	0.146	-0.010	0.131
	(0.279)	(0.106)	(0.137)	(0.142)	(0.129)
New York	0.218	-0.148	0.072	-0.063	0.093
	(0.246)	(0.094)	(0.121)	(0.125)	(0.114)
North Carolina	0.491	-0.079	-0.031	0.012	-0.007
	(0.262)	(0.100)	(0.129)	(0.134)	(0.122)
Rhode Island	0.445	-0.193	-0.298	-0.466	-0.083
	(0.533)	(0.203)	(0.263)	(0.272)	(0.247)
South Carolina	0.289	-0.195	0.017	-0.259	-0.305
	(0.316)	(0.120)	(0.156)	(0.161)	(0.147)
Texas	0.211	-0.029	0.078	0.037	0.098
	(0.238)	(0.090)	(0.117)	(0.121)	(0.110)
Virginia	0.229	-0.023	0.197	0.022	-0.064
	(0.270)	(0.102)	(0.133)	(0.137)	(0.125)
Observations	4,000	4,000	3,999	4,000	4,000
R^2	0.192	0.014	0.036	0.009	0.008
Adjusted R^2	0.188	0.009	0.031	0.004	0.003
Residual Std. Error	2.527 (df = 3978)	0.960 (df = 3978)	1.245 (df = 3977)	1.287 (df = 3978)	1.172 (df = 3978)
F Statistic	44.997*** (df = 21; 3978)	2.777*** (df = 21; 3978)	7.163*** (df = 21; 3977)	1.811 (df = 21; 3978)	1.519 (df = 21; 3978)

Notes: Linear regressions. Alabama is the reference state. *p<0.005; **p<0.0025; ***p<5e-04
